# Tai Chi for Chronic Pain Conditions: A Systematic Review and Meta-analysis of Randomized Controlled Trials

**DOI:** 10.1038/srep25325

**Published:** 2016-04-29

**Authors:** Ling Jun Kong, Romy Lauche, Petra Klose, Jiang Hui Bu, Xiao Cun Yang, Chao Qing Guo, Gustav Dobos, Ying Wu Cheng

**Affiliations:** 1 Yueyang Hospital of Integrated Traditional Chinese and Western Medicine, Shanghai University of Traditional Chinese Medicine, Shanghai, China; 2 Australian Research Centre in Complementary and Integrative Medicine (ARCCIM), Faculty of Health, University of Technology Sydney, Sydney, Australia; 3 Department of Internal and Integrative Medicine, Kliniken Essen-Mitte, Faculty of Medicine, University of Duisburg-Essen, Essen, Germany

## Abstract

Several studies reported that Tai Chi showed potential effects for chronic pain, but its role remains controversial. This review assessed the evidence regarding the effects of Tai Chi for chronic pain conditions. 18 randomized controlled trials were included in our review. The aggregated results have indicated that Tai Chi showed positive evidence on immediate relief of chronic pain from osteoarthritis (standardized mean difference [SMD], −0.54; 95% confidence intervals [CI], −0.77 to −0.30; P < 0.05). The valid duration of Tai Chi practice for osteoarthritis may be more than 5 weeks. And there were some beneficial evidences regarding the effects of Tai Chi on immediate relief of chronic pain from low back pain (SMD, −0.81; 95% CI, −1.11 to −0.52; P < 0.05) and osteoporosis (SMD, −0.83; 95% CI, −1.37 to −0.28; P = 0.003). Therefore, clinicians may consider Tai Chi as a viable complementary and alternative medicine for chronic pain conditions.

Chronic and recurrent pain, which can be persistent and prevalent, is a common health problem and a major cause of high economic costs in relation to health expenses and job absenteeism^1^,^2^,^3^. Chronic pain typically presents obvious physical and psychological damage for pain sufferers. Physically, chronic pain may decrease the pain threshold making sufferers sensitive to subliminal stimuli, and resulting in symptomatic responses including hypertension, insomnia, and astrointestinal ulceration^4^. Psychologically, chronic pain may cause, for example, emotional disturbances, depression, and social withdrawal^5^. Common causes of chronic pain include osteoarthritis (OA), low back pain (LBP), rheumatoid arthritis (RA), and fibromyalgia. In addition to regular treatments (such as medicine and surgery), complementary and alternative medicine (CAM) has an increasingly important role in ameliorating chronic pain^6^.

Tai Chi, a mind-body exercise therapy, is typically used to manage chronic pain conditions. During Tai Chi exercises, the slow motion and weight shifting may improve musculoskeletal strength and joint stability. Concentration and mindfulness meditation may modulate multiple aspects of health including mood, functions of the immune and autonomic nervous systems^7^,^8^. Several trials have documented that Tai Chi demonstrated positive effects on chronic pain^9^,^10^,^11^,^12^, and some reviews have maintained that Tai Chi showed some beneficial effects on chronic pain^13^,^14^,^15^,^16^,^17^. However, the majority of the studies either paid attention to only one disease^14^,^15^,^16^,^18^, or were only qualitative analyses^16^,^17^. Furthermore, the majority of the reviews did not include Chinese clinical studies of Tai Chi for chronic pain due to the language barrier or limited resources for information retrieval^13^,^15^,^17^.

Therefore, the objective of this systematic review was to assess the evidence regarding the effectiveness of Tai Chi in decreasing pain in patients with chronic pain conditions to determine whether Tai Chi is a viable CAM for chronic pain conditions.

## Methods

### Trial registration

The study was prospectively registered in PROSPERO with the number CRD42014014428.

### Search strategy

The following electronic databases were searched from their inception to June 2015: PubMed, EMBASE, OVID-MEDLINE, Cochrane Library, China Knowledge Resource Integrated (CNKI) database, Wanfang database, and Weipu database for Chinese Technical Periodicals. The following key words were applied: Tai Chi, Taiji, Taiqi, Taichi Chuan, Taijiquan, t’ai chi chuan, shadowboxing, and pain. A manual search was conducted at the library of Shanghai University of Traditional Chinese Medicine. The reference lists of the retrieved articles were screened.

### Study selection

The studies that met the following criteria were included in this review: (1) study design: randomized controlled trials (RCTs); (2) participants with chronic pain conditions (duration of more than 6 months); (3) interventions of interest: Tai Chi exercise; and (4) primary outcome that includes pain.

### Data extraction and quality assessment

Two reviewers independently extracted the data using the predefined criteria. We contacted the primary authors when the relevant information was not reported. The differences were settled by discussing the issue with reference to the original article. For crossover studies, only the data in the first phase were extracted considering the risk for carryover effects. The reviewers paid attention to the immediate effects (immediately after the treatments: up to 1 day) and the follow-up effects (short term: between 1 day and 3 months after the treatments; intermediate term: between 3 months and 12 months after the treatments; and long term: more than 12 months after the treatments) of Tai Chi for chronic pain.

Two reviewers independently assessed the methodological quality of studies that were included in this review, using the Physiotherapy Evidence Database (PEDro) scale, which consists of 11 items with a maximum score of 10 points and a cutoff score of 6 for high-quality studies^15^. A previous study reported that the reliability of the PEDro scale for rating the quality of the RCTs was “fair” to “good” and that it was suitable for systematic reviews of physical therapy studies^19^. Any disagreements were resolved by obtaining the consensus of all reviewers.

### Statistical analysis

For the continuous data, the changes from baseline were used in the meta-analysis. The pain outcomes were presented as the standardized mean difference (SMD) and 95% confidence intervals (CI) because the scales were not consistent across eligible studies. The meta-analyses were conducted based on at least two trials using Review Manager Version 5.0. For the expected heterogeneity, the continuous data were pooled using a more conservative random-effects model. The heterogeneity was assessed using the Cochran Q statistic (P < 0.10, was considered to be statistically significant) and was quantified using the *I*^2^ index (where *I*^2^ > 30% indicated moderate heterogeneity; *I*^2^ > 50% substantial heterogeneity; and *I*^2^ > 75% considerable heterogeneity). P < 0.05 was considered to be statistically significant.

The subgroup analysis was conducted based on different diseases. If the studies had two or more control groups, the following order was used to select the control group: placebo; no treatment; waiting list control; attention control; education; and other active interventions. To identify the effects of Tai Chi for chronic pain of OA, the subgroup analyses were performed according to different control interventions and intervention durations of Tai Chi exercise. The publication bias was assessed using funnel plots.

## Results

### Study selection

**Figure 1:**
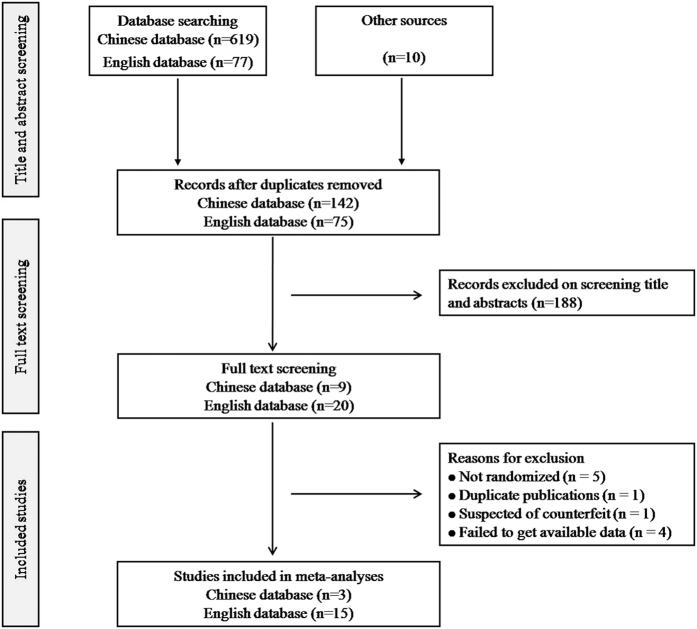
Flow diagram of study selection. RCTs, randomized controlled trials.

A total of 706 records were identified from 7 English and Chinese databases. After removing the duplicates, 217 potentially relevant abstracts were initially screened, and 188 were excluded for failing to meet the inclusion criteria. We retrieved and reviewed 29 full-text articles. A total of 18 RCTs were eligible for this review, including 15 English articles^9^,^10^,^11^,^12^,^20^,^21^,^22^,^23^,^24^,^25^,^26^,^27^,^28^,^29^,^30^ and 3 Chinese articles^31^,^32^,^33^, as indicated by the flowchart in Fig. 1. During the screening full-texts, the studies were excluded for the following reasons: they were not randomized (n = 5)^34^,^35^,^36^,^37^,^38^; they were duplicate publications (n = 1)^39^; they were suspected of being counterfeit (n = 1)^40^; and they failed to present the available data (n = 4)^41^,^42^,^43^,^44^.

### Characteristics of included studies

A total of 1260 individuals with a mean age of 61.43 ± 10.99 years were included in eligible RCTs, which were conducted in Australia, China, Korea, and the USA between 2000 and 2015. The duration of the included studies was between 6 weeks and 28 weeks. The mean ± standardized difference of the therapeutic session and time were 50.44 ± 49.62 (range 10–168 minutes) and 54.72 ± 14.80 minutes (range 20–90 minutes). The follow-up time ranged from 6 weeks to 36 weeks. Of all the studies, 8 RCTs assessed the effectiveness of Tai Chi for OA^10^,^11^,^20^,^21^,^23^,^24^,^27^,^28^, 3 for LBP^9^,^31^,^33^, 2 for osteoporosis^22^,^32^, 2 for fibromyalgia^12^,^26^, and 3 for other diseases^10^,^25^,^30^. Seven used the Yang style^11^,^12^,^21^,^22^,^26^,^29^, 3 practiced the Sun style^10^,^23^,^28^ and 1 used the Wu style^20^. The control groups were conducted in attention control, waiting list control, education, routine treatment control, and other active intervention controls including physical therapy and hydrotherapy. The main characteristics of all included RCTs are shown in Table 1.

### Methodological quality

As shown in Table 2, the majority (94%) of the included trials exceeded the predetermined cutoff score of 6, ranging from 5 to 8 points for OA^10^,^11^,^20^,^21^,^23^,^24^,^27^,^28^, LBP^9^,^31^,^33^, and fibromyalgia^12^,^26^, 6 to 7 points for osteoporosis^22^,^32^, 6 points for herpes zoster^25^, 8 points for RA^29^, and 6 points for stroke^30^. The most common flaws were that the subjects and therapists in all of the trials were unblinded to the treatments, and that 7 RCTs did not perform assessors-blinding^20^,^25^,^28^,^30^,^31^,^32^,^33^. Additionally, allocation concealments were unclear because the detailed allocation procedure was not reported in 8 trials^21^,^24^,^25^,^26^,^30^,^31^,^32^,^33^. The intention-to-treat analysis was rated positive in 12 studies^9^,^10^,^11^,^12^,^20^,^23^,^25^,^27^,^29^,^31^,^32^,^33^. Other items were scored positive in all of the included studies.

### Tai Chi for chronic pain

**Figure 2:**
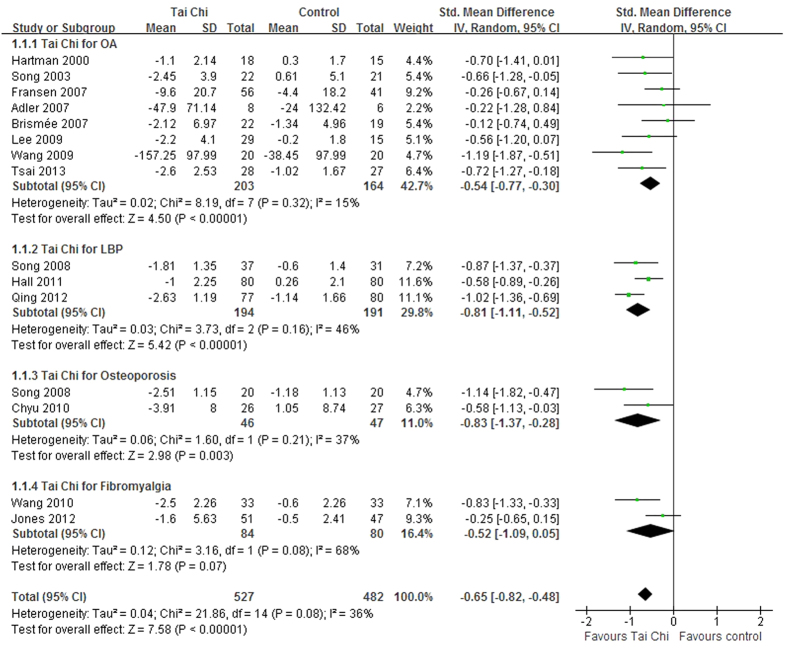
Forest plot of the immediate effects of Tai Chi for chronic pain conditions. OA, osteoarthritis; LBP, low back pain.

The data of a total of 15 studies were pooled in the meta-analysis. The aggregated result indicated that Tai Chi achieved better gains in ameliorating chronic pain compared to the control interventions (SMD, −0.65; 95% CI, −0.82 to −0.48; P < 0.001; Fig. 2).

### Tai Chi for OA

**Figure 3:**
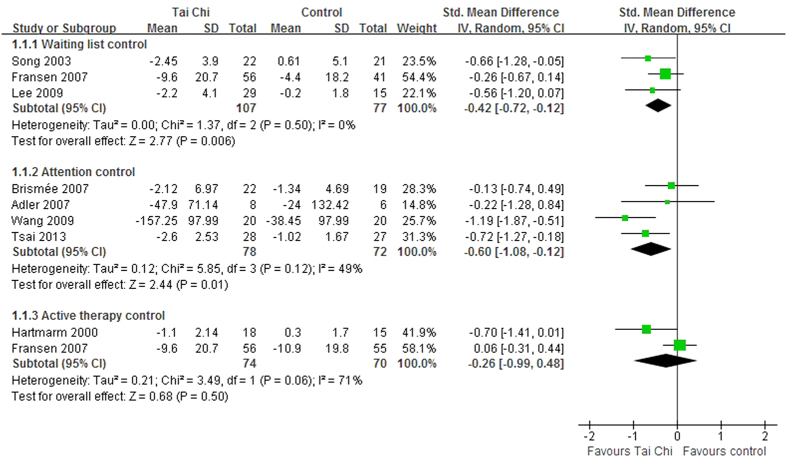
Forest plot of the subgroup analyses of Tai Chi for chronic pain conditions of osteoarthritis based on different interventions in control groups.

The aggregated results of 8 RCTs^10^,^11^,^20^,^21^,^23^,^24^,^27^,^28^ indicated that Tai Chi improved chronic pain in patients with OA compared to the control interventions (SMD, −0.54; 95% CI, −0.77 to −0.30; P < 0.001; Fig. 2). The subgroup analysis was performed to compare Tai Chi with different control interventions. The aggregated results indicated that improvements in pain were greater for Tai Chi than waiting list control (SMD, −0.42; 95% CI, −0.72 to −0.12; P = 0.006; Fig. 3)^23^,^27^,^28^ and attention control (SMD, −0.60; 95% CI, −1.08 to −0.12; P = 0.01; Fig. 3)^10^,^11^,^20^,^21^. However, no significant differences were observed between Tai Chi and active therapy control (SMD, −0.26; 95% CI, −0.99 to 0.48; P = 0.50; Fig. 3)^23^,^24^.

**Figure 4:**
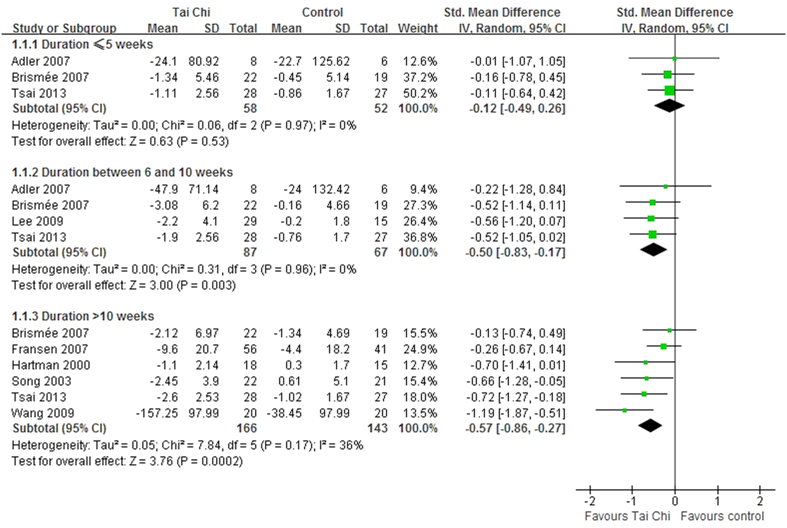
Forest plot of the subgroup analyses of Tai Chi for chronic pain conditions of osteoarthritis based on different durations of Tai Chi exercises.

The subgroup analysis was performed based on different durations: ≤5 weeks, between 6 and 10 weeks, and >10 weeks. For duration ≤5 weeks, Tai Chi did not significantly reduce pain (SMD, −0.12; 95% CI, −0.49 to 0.26; P = 0.53; Fig. 4)^10^,^20^,^21^. However, the Tai Chi group experienced greater improvements in pain for the duration between 6 and 10 weeks (SMD, −0.50; 95% CI, −0.83 to −0.17; P = 0.003; Fig. 4)^10^,^20^,^21^,^27^ and for the duration >10 weeks (SMD, −0.57; 95% CI, −0.86 to −0.27; P = 0.0002; Fig. 4)^10^,^11^,^21^,^23^,^24^,^28^.

**Figure 5:**
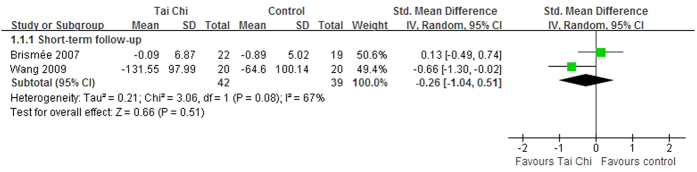
Forest plot of the follow-up effects of Tai Chi for chronic pain conditions of osteoarthritis.

Three trials reported the follow−up effects of Tai Chi for OA chronic pain conditions^11^,^21^,^23^. Two RCTs assessed the short term effects of Tai Chi after a 6-week follow-up^21^ and a 12-week follow-up^11^. Although one of them reported that Tai Chi was effective after a 12-week follow-up, the aggregated results indicated that Tai Chi did not show better short term effects (SMD, −0.26; 95% CI, −1.04 to 0.51; P = 0.51; Fig. 5)^11^,^21^. Wang *et al.* also reported the intermediate term follow−up effects of Tai Chi for OA chronic pain conditions after a 36-week follow-up^11^. Additionally, Fransen *et al.* assessed the short term follow-up effects of Tai Chi for OA, however, the results were ineligible because of inappropriate aggregated results^23^.

### Tai Chi for LBP

Three RCTs tested the effects of Tai Chi for LBP^9^,^31^,^33^. The aggregated results indicated that Tai Chi significantly improved LBP pain (SMD, −0.81; 95% CI, −1.11 to −0.52; P < 0.001; Fig. 2)^9^,^31^,^33^. The Tai Chi durations of these studies were 10 weeks^9^, 24 weeks^33^, and 28 weeks^31^, respectively. One RCT assessed the intermediate term follow-up effects of Tai Chi for chronic LBP, however, the results were not reported^31^.

### Tai Chi for osteoporosis

Two RCTs assessed the effects of a 24-week Tai Chi for osteoporosis^22^,^32^. The aggregated results indicated that Tai Chi significantly reduced the osteoporosis pain (SMD, −0.83; 95% CI, −1.37 to −0.28; P = 0.003; Fig. 2).

### Tai Chi for fibromyalgia

Two RCTs tested the effects of Tai Chi for fibromyalgia^12^,^26^. Although one trial reported better effects of Tai Chi than education and stretching^12^, the aggregated results did not support better effects of Tai Chi in improving fibromyalgia pain (SMD, −0.52; 95% CI, −1.09 to 0.05; P = 0.07; Fig. 2)^12^,^26^. One trial reported that Tai Chi showed better short term follow-up effects for fibromyalgia pain after a 12-week follow-up (mean changes, 2.4 versus 0.7, P < 0.05)^12^.

### Tai Chi for other diseases

Three studies tested the effects of Tai Chi for herpes zoster (postherpetic pain)^25^, RA^29^, and stroke^30^. Irwin *et al.* reported that Tai Chi exercise showed significant improvements in body pain from herpes zoster compared with health education (mean changes, 6.68 versus 3.79, P < 0.05)^25^. The study supported that Tai Chi achieved improvements of pain in patients with chronic stroke compared to general physical therapy (mean changes, 5.55 versus 0.82, P < 0.05)^30^.

One trial reported that Tai Chi significantly improved RA pain compared with attention control (mean changes, 1.00 versus −1.60, P < 0.05)^29^. It assessed the short term follow-up effects of Tai Chi for chronic RA pain and reported that 90% of the patients experienced improvements compared to the baseline in joint pain after a 12-week follow-up.

### Publication bias

**Figure 6:**
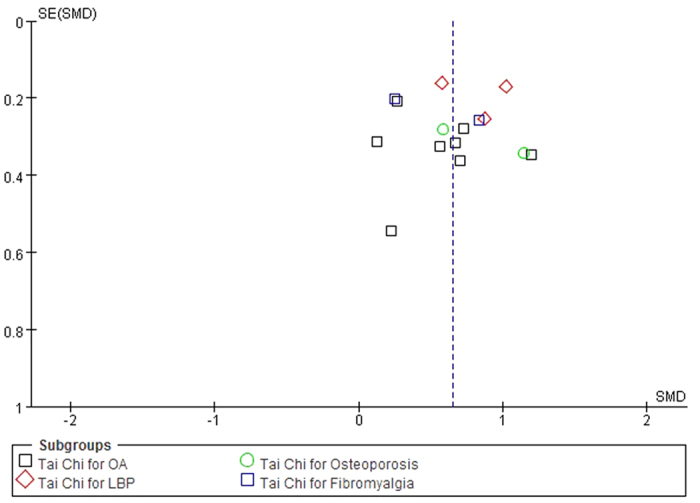
Funnel plot for OA, LBP, osteoporosis, and fibromyalgia. OA, osteoarthritis; LBP, low back pain.

The funnel plots for OA, LBP, osteoporosis, and fibromyalgia were performed including 8 RCTs^10^,^11^,^20^,^21^,^23^,^24^,^27^,^28^, 3 RCTs^9^,^31^,^33^, 2 RCTs^22^,^32^, and 2 RCTs^12^,^26^ respectively (Fig. 6). Regarding the studies of Tai Chi for OA, the publication bias was small because the 8 spots were substantially symmetric. However, caution is advised in interpreting the results of publication bias of LBP, osteoporosis, and fibromyalgia because of a small subset of studies.

### Adverse events

Only 2 studies reported that there were minor adverse events^9^,^21^. One study found sporadic complaints of minor muscle soreness and foot and knee pain at the commencement of the intervention^21^. The other study reported that three participants found a small initial increase in back pain symptoms that were alleviated by the third or fourth week of treatment, and one participant reported an increase in upper back pain that was alleviated once the upper extremity posture had been corrected^9^.

## Discussion

The major purpose of the current review was to evaluate the effects of Tai Chi for chronic pain conditions. The primary finding was that Tai Chi showed improvements in chronic pain for patients with OA, LBP, and osteoporosis. The valid duration of Tai Chi for chronic OA pain might be at least 6 weeks. On the follow-up effects, there was insufficient evidence of the effects of Tai Chi for suffers of chronic pain conditions.

This systematic review assessed the effects of Tai Chi on chronic pain in various common diseases including OA, LBP, RA, osteoporosis, and fibromyalgia. Therefore, a subgroup analysis was performed based on different diseases. The results maintained that Tai Chi showed better effects in improving chronic pain caused by OA, LBP, and osteoporosis, however, there was only moderate evidence of the effects of Tai Chi on chronic pain in patients with OA because other aggregated results were based on fewer eligible studies. Furthermore, the subgroup analysis was performed to compare Tai Chi with different control interventions for chronic pain in patients with OA. The aggregated results indicated that Tai Chi was more effective for participants with chronic OA pain than for those in the waiting list control or attention control groups. However, there was insufficient evidence to support or refute the value of Tai Chi compared with other active therapies because Tai Chi was compared with physical therapy and hydrotherapy in only 2 studies each. Additional, further studies should compare Tai Chi with more active therapies, such as aerobic exercise and acupuncture. Few studies have investigated the follow-up effects of Tai Chi for chronic pain conditions. As for chronic and recurrent pain, more attention should be focused on the long term effects of Tai Chi exercises.

Our results indicated that a minimal valid duration of Tai Chi for chronic pain might be 6 weeks, and the longer duration may achieve better gains. A subgroup analysis supported that 6–10 weeks of Tai Chi significantly improved chronic pain in patients with OA and that long term Tai Chi (12–20 weeks) may be more effective, which is consistent with previous findings^14^. Furthermore, 10–28 weeks of Tai Chi also showed greater improvements in patients with chronic pain of RA, LBP, and osteoporosis. Consequently, long term Tai Chi exercise could be more effective for the management of chronic pain conditions.

Our results are similar to the latest systematic review. Peng’s systematic review suggested that Tai Chi seemed to be an effective intervention in OA, LBP and fibromyalgia, however, it was only a qualitative review including 10 RCTs published between 2000 and 2011^17^. Any qualitative reviews may be problematic because they are often more subjective than quantitative meta-analyses. Two studies were excluded in Peng’s review because one was not a formal published dissertation^20^ and the other used Tai Chi Qigong as the intervention^27^. However, “Tai Chi Qigong” only included Tai Chi exercise. Thus, they were eligible studies for our review. Furthermore, detailed subgroup analyses were performed based on different diseases, control interventions, and durations of Tai Chi. Additionally, the follow-up effects of Tai Chi for chronic pain conditions were focused in our review. Therefore, there was more powerful evidence of Tai Chi for chronic pain conditions in our review.

### Study limitations

There are several limitations in our review: (a) A rigorous search strategy was applied in our review, however, some uncertainty still remains due to bias in location and publication^45^,^46^. (b) Although the predetermined cutoff score of 6 using PEDro scale was exceeded by the majority of studies, there were some flaws in the blinding methods of eligible RCTs. It is difficult to blind the patients and it is impossible to blind the therapists in Tai Chi studies, however, the blinded assessors and concealed allocations should compensate for these flaws. Several trials did not perform these compensated methods. It was suspicious that no participant dropped out during the Tai Chi intervention that lasted for at least 6 months in three Chinese RCTs^31^,^32^,^33^. These flaws may have created potential performance biases and detection biases. Thus, several studies could not be considered to be of high quality. (c) Few eligible RCTs were a major limitation, especially for RA, fibromyalgia, herpes zoster, stroke, osteoporosis, and LBP. Some subgroup analyses were only based on 2 to 3 studies; thus, some conclusions should be interpreted with caution. (d) Our results may be affected by the styles and dosing parameters of Tai Chi such as different styles (Yang-style, Wu-style, etc.) and frequency (number of Tai Chi sessions per week). The eligible studies employed different styles and dosing parameters. (e) Although the pain outcomes were presented as SMD in the meta-analyses, the aggregated results may also be influenced by different outcome measures in eligible studies. Thus, the reliable and valid outcome measure is essential to reduce bias, provide precise measures and perform valid data synthesis. (f) Although fewer adverse events were associated with Tai Chi, definite conclusions are not possible. It can only be assumed that Tai Chi is a treatment option with a low risk of injury.

## Conclusion

This systematic review demonstrated positive evidence regarding the effects of Tai Chi on chronic OA pain, and some beneficial evidences of Tai Chi for LBP and osteoporosis. The minimal valid duration of Tai Chi for chronic OA pain may be 6 weeks, and a longer duration of Tai Chi exercise may achieve more gains. However, there was no valid evidence on the follow-up effects of Tai Chi for chronic pain conditions. There was insufficient evidence to support or refute the value of Tai Chi compared with other active therapies for chronic pain conditions. Consequently, future studies should emphasize high-quality RCTs comparing Tai Chi with other active therapies for chronic pain conditions, and a long term follow-up should be conducted.

## Additional Information

**How to cite this article**: Kong, L. J. *et al.* Tai Chi for Chronic Pain Conditions: A Systematic Review and Meta-analysis of Randomized Controlled Trials. *Sci. Rep.* **6**, 25325; doi: 10.1038/srep25325 (2016).

## Figures and Tables

**Table 1:** Characteristics of included studies.

First authors, year, country	Primary report	Sample size, mean age (year),	Duration weeks	Follow-up weeks	Main pain outcome assessments	Experimental group intervention*	Control group intervention*
Hartman, USA^24^	Lower extremity OA	35 68	12	—	ASE pain; AIMS pain	Yang-style Tai Chi (60 min/24 sessions)	Routine care plus physical therapy
Song, Korea^28^	Knee OA	72 63	12	—	WOMAC pain	Sun-styleTai Chi (20-30 min/36 sessions)	Waiting list
Adler, 2007, USA^20^	Hip or knee OA	14 NR	10	—	WOMAC pain	Wu-style Tai Chi (60 min/10 sessions)	Bingo (More than 8 sessions)
Brismée, USA^21^	Knee OA	41 70 ± 9.2	12	6	VAS pain; WOMAC pain	Yang-style Tai Chi (40 min/36 sessions)	Attention control (40 min/18 sessions)
Fransen, USA^23^	Hip or knee OA	152 70	12	12	WOMAC pain	Sun-style Tai Chi (60 min/24 sessions)	Hydrotherapy (60 min/24 sessions) Waiting list
Irwin, USA^25^	Varicella Zoster	112 70	16	—	SF-36 (Bodily pain)	Tai Chi (40 min/48 sessions)	Health education (40 min/48 sessions)
Song, China^33^	Low back pain	68 42	24	—	VAS pain	Tai Chi plus physical therapy (60 min/144 sessions)	Physical therapy
Song, China^32^	Osteoarthritis	40 62.67 ± 11.2	24	—	VAS pain	Tai Chi plus routine treatment (60 min/144 sessions)	Routine treatment
Wang, USA^29^	Rheumatoid arthritis	20 50	12	12	VAS pain	Yang-style Tai Chi (60 min/24 sessions)	Attention control (60 min/24 sessions)
Lee, Korea^27^	Knee OA	44 69.1 ± 5.4	8	—	WOMAC pain	Tai Chi (60 min/16 sessions)	Waiting list
Wang, USA^11^	Knee OA	40 65	12	36	WOMAC pain	Yang-style Tai Chi (60 min/24 sessions)	Attention control (60 min/24 sessions)
Chyu, USA^22^	Osteoarthritis	61 72	24	—	SF-36 (Bodily pain)	Yang-style Tai Chi (60 min/72 sessions)	Not any exercise intervention
Wang, USA^12^	Fibromyalgia	66 50	12	12	VAS pain	Yang-style Tai Chi (60 min/24 sessions)	Wellness education and stretching
Hall, Australia^9^	Low back pain	160 44.4 ± 13.2	10	—	NRS pain	Tai Chi plus health care (40 min/18 sessions)	Waiting list plus health care
Jones, USA^26^	Fibromyalgia	10 54	12	—	NRS pain	Yang-style Tai Chi (90 min/24 sessions)	Education (90 min/24 sessions)
Qing, China^31^	Low back pain	157 NR	28	24	VAS pain	Tai Chi plus physical therapy (60 min/168 sessions)	Physical therapy
Tsai, USA^10^	Knee OA	55 78.91 ± 7.55	20	—	WOMAC pain	Sun-style Tai Chi (20-40 min/60 sessions)	Attention control (20-40 min/60 sessions)
Kim, Korea^30^	Stroke	22 54	6	—	SF-36 (Bodily pain)	Tai Chi (60 min/12 sessions)	Physical therapy (60 min/12 sessions)

Abbreviations: OA, osteoarthritis; ASE, Arthritis self-efficacy; AIMS, Arthritis Impact Measurement Scale; WOMAC, Western Ontario and McMaster Universities Osteoarthritis Index; VAS, visual analog scale; SF-36, the Medical Outcomes Study 36-Item Short-Form Health Survey; NRS, Numerical Rating Scale; NR: No Reported.

*Intervention/dose: number of intervention time/number of sessions.

**Table 2:** PEDro scale of quality for included trials.

Study	Eligibility criteria	Random allocation	Concealed allocation	Similar at baseline	Subjects blinded	Therapists blinded	Assessors blinded	<15% dropouts	Intention-to-treat analysis	Between-group comparisons	Point measures and variability data	Total
Hartman^24^	1	1	0	1	0	0	1	1	0	1	1	6
Song^28^	1	1	1	1	0	0	0	0	0	1	1	5
Adler^20^	1	1	1	1	0	0	0	1	1	1	1	7
Brismée^21^	1	1	0	1	0	0	1	1	0	1	1	6
Fransen^23^	1	1	1	1	0	0	1	1	1	1	1	8
Irwin^25^	1	1	0	1	0	0	0	1	1	1	1	6
Song^33^	1	1	0	1	0	0	0	1	1	1	1	6
Song^32^	1	1	0	1	0	0	0	1	1	1	1	6
Wang^29^	1	1	1	1	0	0	1	1	1	1	1	8
Lee^27^	1	1	1	1	0	0	1	1	1	1	1	8
Wang^11^	1	1	1	1	0	0	1	1	1	1	1	8
Chyu^22^	1	1	1	1	0	0	1	1	0	1	1	7
Wang^12^	1	1	1	1	0	0	1	1	1	1	1	8
Hall^9^	1	1	1	1	0	0	1	1	1	1	1	8
Jones^26^	1	1	0	1	0	0	1	1	0	1	1	6
Qing^31^	1	1	0	1	0	0	0	1	1	1	1	6
Tsai^10^	1	1	1	1	0	0	1	1	1	1	1	8
Kim^30^	1	1	0	1	0	0	0	1	1	1	1	6

0 = not meet the criteria; 1 = meet the criteria.
